# “Don’t stop believing!” From health religiosity to an equality-enhancing hermeneutic of health promotion

**DOI:** 10.1080/17482631.2018.1555420

**Published:** 2018-12-12

**Authors:** Britta Pelters, Åsa Roxberg

**Affiliations:** School of Health and Welfare, Halmstad University, Halmstad, Sweden

**Keywords:** Health beliefs, health promotion, hermeneutics, religiosity, health disparities

## Abstract

**Purpose**: Health beliefs are usually regarded as subjective understandings of one’s health. They can, however, be re-interpreted by drawing on the understanding that the structural features of the health discourse resemble the characteristics of a religion and on the spiritual dimension of health with its possibly salutogenic influence. The applicability of the notion of “health religiosity” and its consequences for individual health promotion are explored.

**Method**: Data consist of already existent semi-structured interviews. These have been reanalyzed in a deductive-hermeneutical way by using a five-dimensional concept of religiosity as deductive template.

**Results**: The concept of religiosity proved to be productive and revealed that all health dimensions in the case are infused with spiritually ennobled ideas.

**Conclusion**: We conclude that, irrespective of their factual accuracy, the salutogenic potential of ennobled ideas may best be utilized by understanding them hermeneutically. An exploration of a narrative hermeneutic approach to individual health promotion is suggested as the merging of meaning horizons in a hermeneutic dialogue is expected to increase awareness of spiritualized aspects of health beliefs. This may mitigate healthism and health disparities. Moreover, three challenges for individual health promotion are anticipated: realizing the situation, recognizing its complexity and resisting a simplistic practical approach.

## Introduction

This paper explores the possibility of eliciting subjective religiously/spiritually ennobled ideas regarding health and the influence of these ideas on everyday life, i.e., a “health religiosity,” as well as the consequences of such a health religiosity in theory and practice within the realm of health promotion. Religious imagery is often used in science, media, economy and everyday life to describe health-related phenomena as meaningful. Telling examples include a wellness company named *The Health Prophet* (http://www.halsoprofeten.se, last accessed 26 February 2017), a newspaper referring to the 5:2 diet as “the new salvation” (Edgren, 2013), a public health scientist calling major risk factors “the Holy Trinity of Risk” (Schmidt, 2010, p. 17) and smokers addressing themselves as “a bunch of sinners” while waiting for a cigarette break, thus considering smoking as their shared sin which they are about to commit (observed in March 2017). It is this metaphoric relationship between religion and health from which the paper originates and the related body of research that this study contributes to.

**Table I.** Examples of the coding process.

Meaning unit	code	Sub-category	Category
I have once read that a woman who had cancer imagined herself to send out small men into her body to destroy the cancer and that she recovered then (…)	Devotion to mental self-defence; fighting cancer (≈ Satanic antagonist) in-situ (inside body)	Private devotion (personal practice)	Ritualistic dimension
(…) and I think that this works. Metaphorically speaking, a lot is possible if you use the power of thought.”	Belief in the power of will as health guarantee Explanatory model strong personality (reminiscent of cancer personality)	Implementing belief	Ideological dimension

When investigated by scholars, however, the connection between personal religiosity/spirituality [r/s] and health is usually associated with the question of whether known adherence to an official faith, belief or spiritual construct promotes health and alleviates disease or not. A considerable number of investigations (e.g., in the fields of mental health, old age and cancer) provide ample clues that r/s has a positive impact on health (e.g., AbdAleati, Mohd Zaharim, & Mydin, 2016; Jim et al., 2015; Zimmer et al., 2016). According to Koenig (2008), this impact is linked to contributions such as personal coping strategies, methods of behavioural control and enhanced social involvement and support. Other studies discuss r/s in relation to attachment theory and its relational establishment of feelings of security (Kirkpatrick, 1992; Reinert, Edwards, & Hendrix, 2009). These also emphasize connectedness and hope as sources of salutogenic effects (Unterrainer, Ladenhauf, Moazedi, Wallner-Liebmann, & Fink, 2010).

Religiosity and spirituality are often aggregated into r/s compounds in these studies, a perspective that is retained here. Gallardo-Peralta’s (2017) line of argument is followed, which presents religiosity and spirituality as being “closely linked yet distinct phenomena” (Gallardo-Peralta, 2017, p., 1499). With reference to Koenig (2004), religiosity is described as a basically social experience in communities in which beliefs, ritualistic behaviours and dogmata are used to distinguish in-group from out-group. In contrast to this, spirituality can be understood as an individual experience connected to specific, personal beliefs of any kind or, as the *Oxford Dictionary* describes it, as “The quality of being concerned with the human spirit or soul as opposed to material or physical things” (https://en.oxforddictionaries.com/definition/spirituality, last accessed 1 July 2018). Religiosity and spirituality are considered to be linked, as they may enhance each other in collective experiences of shared (established and newly acquired) personal beliefs, behaviours, knowledge, etc. (Gallardo-Peralta, 2017). These latter phenomena are focused upon later on in the study as dimensions of religiosity according to Glock and Stark (1965). The concept of “spiritual health” according to the World Health Organization [WHO] appears to confirm the r/s interlinkage. The WHO (2002) suggests investigating spiritual, religious and personal beliefs together so as to determine degrees of spiritual health, thereby marking those beliefs as linked and hard to separate.

Moreover, it should be noted that the field of r/s is apparently permeated with the concept of “belief.” The term *belief* can be defined as “a mental attitude of acceptance or assent towards a proposition without the full intellectual knowledge required to guarantee its truth” (Encyclopedia Britannica, https://www.britannica.com/topic/belief, last accessed 29 June 2018). Following this definition, a *particular* belief may be understood to be a part of religiosity—more precisely, to be a part of religiosity’s ideological dimension (Glock & Stark, 1965). The term *belief*, however, has been used linguistically as a synonym for *religiosity* as well (Holdcroft, 2006), which means that being religious is regarded as being equivalent to believing. In this linguistic usage, *belief* is often combined with a certain type or focus of belief to form a specific *comprehensive* belief in which all dimensions of religiosity could be affected, such as a Hindu belief. (See also https://en.oxforddictionaries.com/definition/belief, last accessed 1 July 2018.) Because a particular belief may have the potential to affect all other dimensions of religiosity (practice, morality, experience and knowledge; see Glock & Stark, 1965)—i.e., affect belief in a comprehensive sense—these two notions of “belief” are considered to be intertwined. The linguistic diversity will necessarily be reflected in this text, and the reader is called on to be aware of the different notions of belief when reading.

As initially indicated, this empirically oriented research on r/s and health is complemented by another line of research that draws on the metaphoric relationship between religion and health. Medicine, public health and health discourse have been discussed in these studies and are partially or fully confirmed as religion (e.g., Drew, 2014; Pelters & Wijma, 2016; Vanderpool, 2007; Wardlaw, 2011), thus declaring the existence of a “health religion” with shared general structural characteristics in health discourse and religion (Pelters & Wijma, 2016). In this study, we intend to explore whether there are practicing “health believers” in this “health religion” whose beliefs (in a comprehensive sense) can be advantageously described by the concept of religiosity as a personal take on that religion. We will thus investigate what happens if the transcendent meaning of the term *belief* is put back into the term *health belief* as a subjective understanding of one’s health.

Declaring the existence of a health religion may, however, also have consequences regarding the theory of health and the practice of individual health promotion. Given the ingredients of this health religion, such as a comprehensive worldview, moral values or symbols (Vanderpool, 2007), one can assume that health religion resembles the WHO’s understanding of spiritual health as “a phenomenon that is not material in nature but belongs to the realm of ideas, beliefs, values and ethics that have arisen in the minds and conscience of human beings, particularly ennobling ideas” (WHO, 1984).

These mentally elevated or ennobled “ideas, beliefs, values and ethics” (called *ennobled ideas* below) are understood to represent the meaning of *spiritual* in the term *spiritual health*, according to the WHO. This spiritual health—in combination with mental, physical and social well-being—corresponds to the WHO’s health-defining dimensions. Ennobled ideas are regarded as playing a part in inspiring health ideals that contribute to actions for health. Moreover, all health workers are required to act in socially attuned ways so as to contribute positively to people’s health and healthy lives (WHO, 1984) from a caring and holistic stance. The goal is to promote health in all of its dimensions by a “process of enabling people to increase control over, and to improve, their health” (WHO, 1986, p. 1).

This description of spiritual health indicates two challenges. First, based on the conceptualization of ennobled ideas as separate from health or health-stimulating ideals, ennobled ideas are ascribed only an indirect health potential. A qualitative difference thus arises between the spiritual and other dimensions of health, which is supported by a paradigmatic difference. Most health dimensions refer to a reductionist and objectivist biomedical paradigm, while the spiritual dimension draws on a complex holistic paradigm (Chuengsatiansup, 2003). If health religiosity proves meaningful and a direct influence of health beliefs can be identified—e.g., via their influence on other dimensions of religiosity, such as spurring practices and moral judgments, or by guiding the search for and interpretation of knowledge and experiences (cf. Glock & Stark, 1965)—the notion of a separation between an “indirectly influencing” health potential of subjective, ennobled ideas and the “directly influencing” potential of objective health aspects could be questioned. This could finally imply consequences for the paradigmatic basis of health theory.

Second, regarding practice, the promotion of all kinds of health may then become a demanding task due to its increased infusion with ennobled ideas and thus with complexity. That situation may be further complicated if ennobled ideas do not support health ideals. Discrepancies occur given the requirement for socially attuned health promotion that health workers are supposed to meet.

Since the still-seminal Ottawa Charter (Naidoo & Wills, 2016; WHO, 1986), health promotion has been considered one of the major strategies for the individual betterment of health. Despite the charter’s explicit highlighting of the role of environmental and especially social determinants of health, an individual focus has prevailed in neoliberalist societies (Ayo, 2012), which has thwarted the charter’s intention to counteract healthism. Healthism can be defined as “the preoccupation with personal health as a primary […] focus for the definition and achievement of well-being; a goal which is to be obtained primarily through the modification of life styles” (Crawford, 1980, p. 368). It is linked to a moralizing way of judging people’s health and is highly topical today (Powroznik, 2017). The difficulty in counteracting healthism, however, is not surprising, as health promotion is intended to increase people’s health-related control. Health promotion is thus working towards the same goal that healthism is striving for (Crawford, 2000). This poses a considerable predicament, as healthism is thought to constitute a barrier for an empowering, blame-free and truly health-developing health promotion (De Leeuw, 2007). Thinking in terms of religiosity potentially improving health promotion, as in investigating the (dis)continuities between the different dimensions of religiosity, could contribute to a deeper understanding and awareness of the complexity of health in general and to the role of morality in particular, as is indicated by healthism.

## Aim

The aim of this study is to explore the notion of health religiosity and its consequences for individual health promotion as well as the concept of health. The following two questions will be answered:

- Is personal health religiosity describable?
- Does this religiosity perspective reveal new insights into subjective health beliefs that are of practical and conceptual relevance to health promotion?

## Method

### Design

Denzin and Lincoln (2011) present an understanding of “[t]he province of qualitative research” as “the world of lived experience, for this is where individual belief and action intersect with culture” (Denzin & Lincoln, 2011, p. 2). As health and beliefs are major parts of lived experience, we have drawn upon a qualitative paradigm to conduct the research.

A case study was chosen in order to base the investigation on extensive, in-depth, multifaceted data from which a certain life practice in all likelihood emerges. In accordance with the German structuralist notion of structural (not statistical) generalizability, the emergence of a life practice is regarded as proof of its social acceptance as an acknowledged alternative for dealing with a certain task; here, forming one’s health belief (Oevermann, 2002).

The data have previously been analyzed in an in-depth case reconstruction (Pelters, 2012). This former study (approved by XXX, *correct name will be included after revision*) aimed at understanding the construction of health beliefs in women who had been diagnosed as “BRCA-positive” (BRCA stands for “breast cancer genes”) by investigating their health-related life practices. People diagnosed as BRCA-positive are healthy but at high risk for developing hereditary breast and ovarian cancer in the future due to the genetic variation in their breast cancer genes (Kobayashi, Ohno, Sasaki, & Matsuura, 2013). They were therefore offered the opportunity to participate in a medical control regime performed at specialized clinics in Germany so they could interpret their vulnerable health status as healthy, unhealthy or something else. The data were thus collected for a similar research aim and are assumed to be suitable for our analysis. However, the concept of religiosity represents a new, explorative perspective, the application of which is expected to differentiate this analysis sufficiently from the former to reveal new insights (Heaton, 2004).

### Data

Lisa Schall, the focus person of this case study, is the youngest of four women in the Schall-Brause family who are all diagnosed BRCA-positive. The Schall-Brauses are members of the well-educated middle class and have a regionally acclaimed ambition and progress orientation. Nine cases were surveyed and three analyzed in the original study. Lisa was chosen here because her background as a 23-year-old biology student characterizes her as relatively unlikely to adhere to irrational thinking and simultaneously likely to have high health literacy (Sykes, Wills, Rowlands, & Popple, 2013). Both likelihoods support a non-religious approach to health and present her as well-informed and health-aware. Traces of health-religious beliefs would therefore be deemed to emphasize the importance of those beliefs and of the religiosity perspective in health even in this younger, health-aware generation. Moreover, Lisa could be characterized as fairly healthistic. Healthism is a prevailing and discourse-shaping phenomenon (e.g., Powroznik, 2017), and it thus conveys timeliness to the data from 2007.

The set of data consists of three semi-structured interviews of approximately 90–120 min each. Informed consent was obtained prior to interviewing. Two single interviews were conducted with Lisa (SI-L, all names anonymized) and with her aunt, Anke (SI-A). In addition, one family interview (FI) was conducted with all of the female family members who are diagnosed as BRCA-positive (Lisa, her mother Lydia, aunt Anke and grandmother Johanna). These interviews were conducted at Lisa’s (SI-L), respectively Anke’s home (SI-A, FI). The interviews were characterized by openness and highly congruent statements between the different interview formats. They were based on a thematic interview guide which addressed genetic testing and counselling, science/genetics, health and body and which focused on gaining information about experiences and understandings regarding these topics. These interviews are made available as verbatim translated texts. All data can be obtained from the first author.

### Analysis

The data were reanalyzed with a hermeneutic stance and a deductive approach before the new case description was compared to the existing case reconstruction in Pelters (2012).

First, the data were read through several times, and the parts of the text relevant to the aim of the study were chosen as the study sample.

Second, the chosen parts were coded in a process informed by deductive content analysis (Elo & Kyngäs, 2008). Glock and Stark’s (1965) dimensions of religiosity (see below) were used as a structured analysis matrix for deductive coding. Each of the five dimensions represents one coding category. Corresponding meaning units (i.e., text sections of different sizes from words to sentences) were assigned to these categories during the analytic process. The mentioned sub-specifications of the five dimensions are regarded as sub-categories and were treated accordingly: i.e., meaning units were assigned to them, and all existing sub-categories were finally gathered under the “main heading” of the respective dimension (see example in Table 1). During the process of sorting out the text sections that correspond to each (sub-)category/dimension, a hermeneutic stance was adopted (Oevermann, 2002). This hermeneutic stance comprises an interpretation that aims at the text’s latent meaning, as it applies an understanding of religiosity to an originally non-religious text. This implies that the text needs to be interpreted at the level of what it talks about figuratively rather than settling for what it literally says (Graneheim & Lundman, 2004). The “manifest stage” of the usual analytic process in content analysis is mostly leapfrogged here in support of the “latent stage” of building themes. The coding process of the meaning units is then based on this new, latent layer of meaning. The result of this deductive-hermeneutic analysis is a new case description which is supported in this text by quotations originating from Lisa (if not stated otherwise).

The third comparative step is carried out by contrasting the main result of the new description and the old reconstruction in search of similarities and differences between the two adaptations of the same data.

### Glock and Stark’s concept of religiosity (1965) as a theoretical tool for re-analysis

Glock and Stark’s (1965) concept of religiosity has been chosen, as its five-dimensional classification is thought to provide an applicable and more differentiated tool for investigation compared, for example, to Allport and Ross’s extrinsic/intrinsic model (1967) or Lenski’s four-dimensional model (1963).

- The ideological dimension of faith describes what is believed, divided into *warranting, purposive* and *implementing beliefs*.
- The ritualistic dimension of outward practice describes how religiosity is practically expressed *publicly* and *in private*.
- The experiential dimension of adventure includes emotional religious experiences ranging from the *simple experience* of sensing a presence to more *qualified experiences* such as the feeling of practicing the will of a transcendent power.
- The intellectual dimension of knowledge includes the possession of *theoretical knowledge* and the *ability to present it correctly*, according to the authority.
- The consequential dimension of religious effects describes *morally justified behaviors and attitudes* (determining and equaling “proper conduct”) and thereby the ethical impact of religion on everyday life.

The concept appears, moreover, to be established, as it has been used in a variety of studies, ranging from investigations of religious practice and socialization (Anthony, Hermans, & Sterkens, 2007) to investigations of issues such as happiness (Amalia, Riani, & Julia, 2016) or fraud prevention in the workplace (Purnamasari & Amaliah, 2015) to investigations of trust in marketing (Minton, 2015). Due to its inclusion of an ideological dimension of belief, which is interpreted as resembling spirituality, the concept appears to be consistent with aggregating r/s.

It should be noted that Christian concepts are used as heuristic metaphors to present spiritualized aspects of the case in the results section. This is deemed feasible, as both the geographic connection of the data and the health religious setting (Pelters & Wijma, 2016) point to Christianity as a relevant cultural context.

## Results

In this section, Lisa’s health religiosity is described by using Glock and Stark (1965) dimensions of religiosity.

### The ideological dimension

A warranting belief states the existence of a transcendent entity. In Lisa’s case, this entity is “the power of nature” (FI), which she claims to believe in. To understand the warranted core of the transcendent entity, it is necessary to see what that power of nature comprises as its crucial characteristic. This characteristic may be elicited by looking at the episode of “the moon calendar”:“

Lisa: I will choose a good date [for the risk-reducing oophorectomy] then.

Anke: Moon calendar.

Lisa (addressing Anke): Yes sir, will do, you can choose it for me when it is time.

Interviewer: Why moon calendar?

Anke: Because I had both my operations according to the moon calendar and both have been completely free of pain and gave a super result.

Interviewer: What does that mean, using the moon calendar?

Anke: There must be something about it. (…)

Lisa: I am nature-believing, I believe in the power of nature, but not that super esoterically. (FI)

The moon calendar is introduced here as a device that can foretell the way of the power of nature. Lisa intends to use it to determine a date that will guarantee a successful risk-reducing oophorectomy. As “successful” translates into “securing prolonged health and the absence of post-operative complications,” the advancement and result of the event point to health as the actual core of nature. There is thus something like a given health potential that is actualized by its (natural) forces: i.e., a basic “health nature” as a divine entity.

For Lisa, the health nature materializes itself in humans as their genes. She describes genes as “something that (…) is simply there in our bodies” (SI-L), immutably “constitutes us” (SI-L) and determines the development of cancer: “I know what cancer is and thus have an advantage; I understand inheritance and how it works” (FI). Genes are thus understood to be health-determining agents that cannot be altered or dismissed. Lisa’s way of defining genes can be regarded as a purposive belief in genes as mediators of a health potential. The genetic potential is the purpose determined by the ways of the health nature at whose “mercy” Lisa is.

The divine purpose includes, in Lisa’s case, an ordeal that requires mastering the challenge to secure her health in the face of her “enemy” (FI)—cancer—which “has always been there” (FI) in her family. “Know your enemy” (FI) is thus her requirement for being able to fight it. Her body becomes a sort of battlefield here on which she has to be physically better/faster/stronger than the cancer—e.g., by training in karate with the aim of doing it “for [her] body and against the cancer” (FI) or by reflecting on the mental tool of sending out “small men into [her] body to destroy the cancer” (SI-L). The motivation for these practices consists in the explanatory model of health risks (which perpetuates the necessity for preventive efforts) and the explanatory model of the strong personality. According to the latter, not only deeds but also a willing attitude—Lisa’s “power of will” (FI)—are required. This explanatory model appears to cite the obsolete explanatory model of the “cancer personality,” i.e., the idea that certain personality flaws promote cancer (Scholz, 2011), and avert the relevance of this explanatory model. Both explanatory models determine proper (i.e., morally right) conduct to ensure health, to bring out the best in the genetically materialized health potential and to reach a beneficial agreement with the health nature. These models deliver the conductive means by which the health purpose is realized and thus represent implementing beliefs.

The function of Lisa’s health belief is to support the active, “gladiatorial” defence of health against the threatening cancer enemy (who could be positioned as the “Satanic” antagonist). Simultaneously, she has to deal with her conviction of not being able to win against the overwhelming, potentially fatal power of the health nature materialized in her genes (“I am prepared for getting it probably” [FI]). Her agreement with this health nature is thus rather fragile and demands a constant commitment to health activities in an ongoing “David-versus-Goliath” showdown.

### The ritualistic dimension

In accordance with Lisa’s particular belief that she is able to actively influence her health, the ritualistic practice dimension is very important for Lisa. The centrepiece of Lisa’s private devotion is her body, which needs to be kept clean from cancer in order not to disturb the way Lisa has arranged her life. Her statement: “I eat healthy, I don’t smoke either” (FI) points at practical strategies aiming at the mastering of typical risk factors. After the BRCA test, these strategies were complemented by doing karate in anticipation of and as a staging of her fight against cancer, thereby mediating the feeling “I can fight it [cancer risk]” (FI). The self-disciplining and self-educating strategy of telling herself “no, you have to go [and exercise] for your body and against the cancer” (FI) is kept up even when Lisa is unmotivated. The strategy is used in a preventive and curative context, with the latter being represented by mental preparations to meet and fight cancer *in situ*:

I have once read that a woman who had cancer imagined herself to send out small men into her body to destroy the cancer and that she recovered then, and I think that this works, metaphorically speaking. A lot is possible if you use the power of thought. (SI-L)

Besides this private devotion, Lisa also regularly practices public rituals. Here, the biannual medical cancer screening is worth mentioning. These visits to the cancer clinic resemble sacred ceremonies in that they are characterized by a strictly schematized course of events:

Lydia: We leave at 6 a.m. and are in X-town at 8 a.m. Then the gynaecological examinations start, one after the other, and then it is time to quickly go to the university. They have a cafeteria where we eat, and at 2 p.m. we have the appointments for the breast consultation. (FI)

The ritual is performed by initiated people (doctors, cf. Wardlaw, 2011). Given her private ritualistic devotion to fighting and cleansing practices and her ideological beliefs (see above), the visits are intended to prove if Lisa still “walks under the grace” of the divine health nature. The negative screening result functions as a proof and “absolution.”

Moreover, Lisa’s familial position as the biology-studying health expert and contact person for the clinic makes her a sort of semi-initiate, which includes a licence to judge (see the example concerning her mother in the “consequential dimension” section) and educate others. This prerogative is represented in her aunt’s reaction to the question regarding how she, the aunt, would describe a gene that confirms Lisa’s expert position by addressing her as knowledgeable: “[I]f I would have to describe a gene, I would call Lisa and say, ‘Lisa, there is someone who wants to know what a gene is. Tell him’” (SI-A). Lisa could thus easily regard herself as being in charge of health, even in her family as part of her public practice, which simultaneously confirms her competence of staying ahead of the cancer enemy.

Lisa’s different practices fulfil the function of boosting body, mind and social authority as a health-service ritual and a defence against any lurking bio-psycho-social weakness which might let cancer slip in.

### The experiential dimension

Lisa is first and foremost having simple yet ambivalent experiences of a divine presence in her life. On the one hand, Lisa experiences satisfying effects when she exhibits proper health conduct. For example, she obtains “a great deal of self-assurance,” “power” and “the joy of fitness” (all SI-L) from her karate training, an exercise which she started after the announcement of her positive BRCA-gene test result. These satisfying effects could be interpreted as a reward from the health nature and thus as a sign of its presence in Lisa’s life. As a consequence, feelings of hope, trust and faith concerning the justice of that divine entity are emotional experiences that Lisa may have when facing health issues.

On the other hand, Lisa is also experiencing strong feelings of fear, sometimes accompanied by resignation and anger, when confronting health issues.

[W]ell I always think, the worst case for me would be when I get pregnant and get ovarian cancer at the same time […] this would be the worst case, because then I would have to decide […] if I now want the child or want to live or something like that. (FI)

The quotation shows that her own future and that of her family is inextricably intertwined and overshadowed by the cancer threat waiting in her genes. The health nature is thus also experienced as a punishing deity who needs to be appeased by acting and thinking in the right way. The balance between a punishing and a rewarding deity, however, is quite a delicate one and appears to depend on Lisa being able to keep up the good mind-body work.

Beyond these simple experiences, she sometimes even experiences herself in a qualified way as an instrument of the divine by informing others about health topics or by helping them to suffer less. One example of this occurred when Lisa informed her mother Lydia of the BRCA-test result despite Lydia’s unwillingness to know. Another example occurred when Lisa talked Johanna into participating in the family interview despite Johanna’s doubts because she wanted to help ease her grandmother’s burden of suffering: “I would have liked that she talked about it [cancer experience] but she has a very hard time talking, as you [the interviewer] surely have noticed [during the family interview]” (SI-L).

Experiences like these appear to reveal a “missionary zeal” that makes Lisa believe she is entitled to ignore personal boundaries and choices because she experiences herself as acting responsibly with life-saving intentions.

In the experiential dimension, the punishing and rewarding deity functions as a strong motivation for saving herself and others and induces a sense of mission and a shifting balance between hope and fear.

### The intellectual dimension

Lisa presents herself as the knowledgeable expert due to her studies in biology and claims to know “what cancer is” and to “understand inheritance and how it works” (FI). This allows her to use knowledge to ease the burden created by the genetic load for both herself and others. One such incident occurred when Lisa tried to reduce her grandmother’s feelings of guilt about passing on the genetic variation by informing her of the contingency of inheritance. At the same time, the accuracy and amount of her knowledge could be problematized from a pedagogical point of view (Dale, 1969), as Lisa has stated that she lacks the actual experience of meeting someone with severe cancer. Moreover, she did not learn more than necessary about genetics because it seemed boring to her. Her theoretical and experiential knowledge can therefore be described as limited and possibly faulty, yet it still exceeds that of her family.

Intellectually, Lisa finds herself in a delicate balance between knowing and not knowing. This balance, with its emphasis on presenting understanding, functions as an amplifying justification, ensuring her expert position in the family, her initiated self-image and her way of dealing with and understanding her genetic state. This state does not provide a reason for doubts or uncertainty. Lisa’s way of acting and thinking becomes inevitable.

### The consequential dimension

It can be observed that Lisa lives her life in the spirit of health as she emphasizes that she is doing health-related activities with the right attitude: i.e., a consciously health-related one.

Lydia: My husband and I, we go to the gym (…) We are Swabians, it costs a lot of money and then you really have to do it (…) but afterwards it feels great. But it’s really nice.

Lisa: But I’m going there [karate training] consciously because I tell myself I have to do something for my body, not because I think “ok, I just go there now” but I am really doing it consciously. (FI)

As she points out her right attitude as a response to her mother’s description of going to the gym in part because it costs so much and feels nice, her statement can also be regarded as an indirect judgement of her mother’s “faulty” attitude. Driven by this attitude, she regards obedience to the rules of proper conduct and belief as her responsibility, which also allows her to outmatch others by judgement. These others include her mother, who does not address health issues with the “right” spirit.

Two rewards come together with this responsibility. On the one hand, she matches the family’s demands for active health performance and a disciplined self-presentation which guarantees familial inclusion and support (as seen in the comparison of the not-well-liked grandfather, Herbert, who is considered to publicly “suffer from every disease” [FI] in contrast to the beloved grandmother, Johanna, who does not engage in “whining” [according to Lydia, FI]). On the other hand, she strengthens her social position as an exemplary authority, who has the right to judge and educate others of the consequential way of the “righteous” (e.g., by educating her grandmother about genes, as mentioned above). Proper health conduct is thus awarded with social embedding and a beneficial social position. This might be lifesaving in a family that tackles health challenges collectively (as e.g., is represented by going on a “family trip” [FI] to get a biannual cancer screening) and exercises a great amount of social control.

On the consequential level, her enlightened conviction adopts the function of labelling and securing her health-conscious behaviour as morally right, and it justifies social support, control and acknowledgment.

## Discussion

The aim of the study was to explore the applicability and utility of the notion of health religiosity and its practical and conceptual consequences for health and individual health promotion.

### Applicability and added value of the health religiosity perspective

Lisa’s health belief shows how different dimensions of health are infused with “rightful” religiosity due to emerging ennobled ideas. In regard to physical health, Lisa believes in a genetically mediated divine power of nature that includes health potentials and risks. As concerns mental health, her ideas of a power of will, linked to a strong personality and personal responsibility, can be emphasized. Regarding social health, Lisa considers herself to be in the position of a semi-initiated expert who administrates social responsibility as a “missionary instrument” of the divine so as to support and control familial others who are not quite as knowledgeable. It is this combination of “rightful” practices and attitudes that grants her security, “salvation” and ultimately a good life free from cancer and with a chance for self-actualization. Different health dimensions that are at present framed as non-spiritual—i.e., as dimensions that are or should not be merged with ennobled ideas—become clearly “spiritualized” by adding a spiritual quality despite a lack of ideas connected to traditionally religious ideas or spiritual constructs. Given these results, and drawing on the notion of structural generalization (Oevermann, 2002), it can be concluded from Lisa’s case that it is possible to apply a subjective religiosity perspective and understand health-related convictions and practices in terms of religiosity within a health religion. But is it useful?

In order to determine the benefit of the perspective, the result is discussed in relation to a summary of the case reconstruction in Pelters (2012): Based on available explanatory models of cancer, BRCA and DNA, Lisa secures social presentability by the performative means of fighting and competition. This turns her way of living into a very health-conscious one. Lisa becomes the health expert of the family, granting her authority and reassurance within the family. Being 23 at the time of the interview and at a critical life junction, as well as believing in the available familial interpretations regarding cancer vulnerability, are factors that for Lisa add up to her socially assigned cancer vulnerability and her genetically enhanced drama of adolescence.

This perspective appears at its heart to be down to earth and to point to an attitude of active coping rather than of struggling with giants or gods. Family relations are constantly present but appear as a background for self-presentation and self-configuration. Moreover, Lisa’s biographic position is emphasized as important for understanding the case. Compared to this, the religiosity perspective as presented in the result section shows three differing features.

First, it disregards the biographic dimension and turns the struggle for “salvation” into a permanent ordeal.

Second, the perspective pronounces a moralized sense of “mission,” “righteousness” and “superiority,” all of which support Lisa’s position as an enlightened health expert, as a cooperation partner of the divine health nature and as a familial social authority. This position implies a double influence: first, Lisa assigns moral justification to her own health commitment as a guideline for everybody else’s health behaviour; second, Lisa exerts social support, control and acknowledgment so as to save other family members. Personal certainty and knowledge are key to convincing others of her expertise. Otherwise, Lisa’s position and her whole health approach would be challenged, as both are founded on her ideological base of ennobled ideas. The social aspect changes its connotation from being individually meaningful to being an explicit and essentially moralized social “mission” with individual repercussions. Her family relationships advance from being a background to being a vital space for Lisa to prove her capability and moral health liability by “preaching her health gospel.”

Third, her understanding and practice are charged with emotional intensity. She believes in the overwhelming power of a punishing or rewarding health nature that demands health services and willpower for “salvation.” This particular belief and the ever-present “Satanic” cancer enemy turn her reality into a constant “doomsday” situation characterized by a “gladiatorial” health defence, a fragile agreement and a hierarchic relationship with the deity. This relationship could be deemed an anxious and ambivalent attachment, “characterized by the participant’s being uncertain about the availability of the attachment figure, perceiving the person as inconsistent and alternately warm and cool” (Reinert et al., 2009, p. 105). Hope and despair can alternately be aroused.

Such a deified description could be regarded as a simple rhetorical trick that elicits the emotionalized image it pretends to merely describe. This is, of course, true—but then again, not entirely. On the one hand, such descriptions are comprehensible and are applied in various contexts (see initial paragraph), which suggest that they represent an accepted social practice. On the other hand, the religiosity perspective indicates the easily missed facet that Lisa’s struggle between feelings of inferior vulnerability and superior capability is much more intense than it seems on the outside to be. Taken together, the “resistance is futile” impression might be a very common phenomenon.

Moreover, even education may appear to be dangerous from Lisa’s point of view. Despite the fact that Lisa’s knowledge base appears to be less than accurate and consists of demanding concepts, the importance of Lisa’s ideological base of ennobled ideas might make her reluctant to reconsider that base. Questioning her belief in the balance of the power of nature and her willpower may mean disturbing the main salutogenic ground for Lisa’s health-promoting actions. It is the stability of these ideas and their ritualistic, experiential and consequential implications that make her intransigently health-oriented and immune to a change that would imply uncertainty.

The added value of the religiosity perspective thus consists of directing attention to the emotional saturation and moralized relational dynamics ingrained in one’s comprehensive health belief and to her salutogenic contribution to health.

### Conceptual challenges

As we learn from Lisa, the spiritualization of health narratives turns them into existentially meaningful “truths” that should not be questioned to promote health. The assumption of spiritualized narratives matches the salutogenic orientation, which has inspired health promotion for 20 years (García-Moya & Morgan, 2017) and may contribute to a deepened understanding of resources related to meaningfulness, comprehensibility and manageability. However, understanding health in terms of biomedical concepts—a productive approach concerning ill-health and risk prevention—may not be adequate when it comes to healthy health states. Objective health cannot capture health’s subjective, idea-infused entirety and therefore cannot be more than an approximation of its complexity.

This flaw in capturing the phenomenological complexity of health corresponds to the approximate character represented by objective health’s theoretical basis: the reductionist, materialistic paradigm of biomedicine in search of a truth that is independent of human beings. That objective truth will remain partial without the subjective ennobled ideas that are important for shaping health beliefs and behaviours. Chuengsatiansup’s (2003) notion of a complex, holistic paradigm—which emphasizes that the whole is more than the sum of its parts—may provide a wider frame of reference that raises awareness of the complexity and idea-infusion of health. We argue, however, for a social subjectivist paradigm (Berthon, Pitt, Ewing, & Carr, 2002), in particular for the approach of hermeneutics (Mantzavinos, 2016). We regard this approach to be a better-suited vantage point due to its focus on understanding the intersubjectively created meaning that an aspect of reality may adopt for a certain person. This approach appears to be best qualified to uncover and utilize the health-infusing and health-shaping latent ennobled ideas in health promotion by merging meaning horizons through a mutual dialogic sharing of narratives (Svenaeus, 2000). However, health promotion is an area with particular requirements. We therefore suggest that Svenaeus’ hermeneutics of medicine is in need of elaboration.

First, the positions of the involved parties appear to be rather symmetric in comparison with the doctor–patient relation. This fact calls for a thorough investigation of the client’s complex, idea-infused meaning structure. The aim is to prevent the uncritical assumption that the involved parties think in similar ways because they seemingly refer to similar knowledge and experiences.

Second, the goal of the individual health-promotive meeting is not medically defined recovery but a rather fuzzy actualization of someone’s “fullest health potential” (WHO, 1986, p. 1). The latent, prospective quality of the concept “potential” (https://en.oxforddictionaries.com/definition/potential, last accessed 15 March 2017) may, however, result in acting on an assumption that may not be realistic and may thus be harmful to the individual or not aligned with that person’s health belief and therefore potentially inept. Hence, the goal of individual health promotion needs to be thoroughly negotiated and anchored in the actualities of life and health belief, which also accommodate the environmental orientation advocated in health promotion (Naidoo & Wills, 2016).

### Mitigating healthism and health disparities

Introducing a hermeneutic stance to health promotion may mitigate a moralizing healthism as expressed by processes of blaming and shaming the victim, which are known downsides of individual responsibility (Guttman & Ressler, 2001; Ten Have, de Beaufort, Teixeira, Mackenbach, & van der Heide, 2011). These processes may occur in health promotion, yet they may have a detrimental impact on its core values of participation and empowerment (Naidoo & Wills, 2016), as shame translates into feelings of being trapped, powerless and isolated (Brown, 2006) and may result in disconnection from both self and others (Dayal, Weaver, & Domene, 2015). Acceptance and empathy—cornerstones of the hermeneutic process—have been described as promoting shame resilience (Brown, 2006) and a culture of safety with which to deal with shame (Dayal et al., 2015). Exploring a person’s health belief and its surrounding, possibly shame-shaping socio-cultural expectations (Brown, 2006) is considered to be a part of this hermeneutic process. Healthism will thus most likely be reflected upon. This may prevent unaware repetitions of healthistic demands, promote connecting empowerment instead of isolating responsibility and allow for the establishment of allegiances between promoters and clients. It is assumed (Brown, 2006) that this approach works both individually and in groups, which may allow for its application regardless of how health promotion is defined (De Leeuw, 2007).

Healthism may, however, be perceived as a counterargument to this seemingly extended, naive “trust” in people’s health beliefs, as it describes a certain belief in how health can be achieved that could oscillate between being beneficial and being harmful to one’s health (Håman, Barker-Ruchti, Patriksson, & Lindgren, 2016). The phenomenon of “wrong” or “harmful” health beliefs is, moreover, well-known—for example, in the field of screening in which a lack of perceived susceptibility may result in low participation in cancer screening (Guilford, McKinley, & Turner, 2017). Other research on the same topic (Oscarsson, Wijma, & Benzein, 2008), however, provided evidence that women’s reasons for non-attendance are not simply wrong but complex. The latter authors advocate the facilitation of “a co-operative discussion […] to contribute to a mutual understanding” (Oscarsson, Wijma & Benzein, 2008, p. 26) between all parties involved instead of raising awareness of dangers in a correcting fashion as the former authors do. Opting for dialogic mutual understanding matches our suggestion. Please note that we are not recommending a naïve trust in everything people believe in; we are instead recommending a careful, critical and context-sensitive yet respectful hermeneutic exploration of health beliefs.

A hermeneutic exploration of this kind is also deemed suitable to mitigating certain health disparities: namely, those which might be connected to being labelled inferior and wrong. Some clues to the importance of labelling can be collected from different angles: Blaxter (1997), for example, points out that “accounts of health and illness are accounts of social identity” (Blaxter, 1997, p. 747). People are thus unlikely to devalue that identity by approving their own inferiority implied in the term health *in*equality as a label that is applied to the group(s) they belong to. Bromseth and Darj (2010) observe that being the focus of group-tailored empowering interventions conveys, in turn, that the target group is considered powerless as only those without power are in need of empowerment. That (unintended) message could be an assault on the target group’s self-image, which could even make the group members internalize feelings of powerlessness. Broom (2008) says that new subject positions may emerge, which gain their identities by actively resisting health prevention (and promotion) as activities which are characteristic of “paragons of virtue.”

In all of these cases, chances for understanding, let alone change, are diminished. The message of an—implicitly or explicitly—moralized “good” health commitment constituting the healthy subject clearly has the potential to contradict the narratives of those with poorer health. This narrative contradiction is contributed to by both those who are “deviant” (as described) but also by those who are “normal,” as the identity work of the normal, healthy subject calls for keeping a social distance from those who are labelled “unhealthy” so as to avoid the suspicion of being one of “them” (Crawford, 1994). Opening up a dialogue without presuppositions would thus actually mean taking the risk of blurring the social divide, which manifests itself in unquestionable truths about health. It would also mean levelling the interpretative prerogative of those who own that truth. The term *truth* refers here to simple health messages that advocate the importance of a certain understanding of health above other understandings of that complex, yet often simplified matter. This does not mean that there are no health truths, but rather that these truths are far more complex than public health messages such as the “change 4 life” campaign in Great Britain may suggest (https://www.nhs.uk/change4life/about-change4life#ierUytCCYuqFtJk2.97, last accessed 28 December 2017). It is this precise complexity that calls for a situated understanding.

This implies two chances for mitigating health disparities. First, it means resisting an automatic labelling of people’s beliefs as “wrong” (i.e., inferior/bad) or “right” (i.e., superior/good). Resisting this reflex will level the normative disparity between what is regarded as true and false knowledge, as is, for example, represented by the concept of health literacy (see e.g., Smith, Nutbeam, & McCaffery, 2013). This disparity usually comes with a mandate for judging and correcting mistakes and misunderstandings, and it may thus provoke resistance and identity defence. Its overcoming (or at least adjournment) might thus be just what is needed for the hermeneutic exploration of health beliefs as an open-ended process. This may then result in unravelling the salutogenic and pathogenic potential of someone’s health belief in a situated way—for example, with regard to its socio-cultural context and meaning. That positioning may disclose possibilities for change, but it could just as well diminish the need for (correcting) action, as the self-evidence of “the right way” to health may have become more complicated during that process, opening up a safe space by empathetic and respectful exploration. Both change and continuity outcomes could result in mitigating practical, statistically significant health disparities, even if they concern different ways of understanding health.

A possible side effect of this process may incidentally apply to healthy subjects as well. As the truth about health may start to flutter in a situated way, the moralizing power of healthism might in the long run become questionable, thereby opening up possibilities for a more diversified health practice for all. That might just be the butterfly effect of hermeneutic exploration in health promotion.

### Practical challenges

Lisa’s health religiosity elicits three practical challenges for health promotion. First, health workers may altogether miss the health-religious challenge they face if they do not recognize the spiritual infusion. Lisa’s health approach could just as well be seen as a completely secular health-promotive practice, which means missing out on the relational and emotional aspects of her health belief. These aspects need to be seen in the light of the salutogenic effects of r/s (e.g., Koenig, 2008), which imply that not realizing or opposing Lisa’s ennobled ideas may not prove conducive, salutogenically speaking.

A second complication could be provided by the observation that Lisa’s spiritual potential does not seem to be solely health promoting. Her commitment to a ritualistic health practice characterized by (self-)discipline and (self-)education could be just as helpful in a physical sense, as it may be mentally stressful or socially demanding. A person’s health belief may thus have paradoxical effects on different health dimensions, promoting some while endangering others. This challenges a health promotion which aims at realizing people’s complete health potential and requiring health workers to attune to the socially specific variations of their target audiences’ beliefs. Health workers are thus left with the question of how to consider and deal with the whole complex of conflicting health dimensions and situation-complicating ennobled ideas.

The third challenge is connected to health-promotive “business as usual.” When it comes to individual health promotion, the concepts of self-efficacy-oriented empowerment and health literacy often come into play. Based on these concepts, responsibility for health is assigned to an individual who is supposed to understand health information within the normative discursive frames of what “good health” and a “healthy lifestyle” are thought to be. These frames should inform one’s actions (even when applying critical health literacy (Sykes et al., 2013), irrespective of individual understandings of good health (Spencer, 2014). Moreover, the expectation that a desired behaviour will follow from disseminating medically accurate yet tailored information is popular, albeit also widely challenged (Leahy, 2012). This approach is indirectly challenged by Lisa’s health religiosity. Her particular mixture of biomedical and spiritual aspects questions the one-sidedness of a hegemonic narrative and shows the difficulty of generally sorting right from wrong. The challenge is thus not to take the easy way out by turning to that oversimplified “business as usual” approach and dismissing someone’s ennobled ideas too easily.

### Limits of investigation and suggestions for further research

The conclusions of this article are derived from one case, which must limit their scope. Drawing on the aforementioned structuralist generalization (Oevermann, 2002), health religiosity can yet be identified as an accepted social practice. Moreover, the conclusion is consistent with the basic objective of qualitative research: to seek the variation of the research phenomenon so as to exhibit its richness (Dahlberg, 2006), which cannot be achieved by quantitatively guided collection or numerical description.

It is, however, impossible to determine how widespread the phenomenon of health religiosity or its associated challenges are. Further research is needed to study health religiosity, its dissemination, implicit challenges and relationships with different contexts and understandings of health. Further research is moreover needed to elaborate and explore the theoretic underpinnings of a hermeneutics of health promotion.

## Conclusion

The concept of religiosity with its five dimensions can be productively applied to health beliefs, thereby revealing a spiritualization of health dimensions by infusing ennobled ideas. This poses three practical challenges for health promotion workers: to realize the situation, to recognize its complexity and to resist simplicity in health-promotive efforts. On a conceptual level, the results support the social subjectivist paradigm in general and a hermeneutic of health (promotion) in particular.

Such a hermeneutic is proposed to aim at merging different meaning horizons in dialogues that focus on the exploration of the variety of narratives relevant to people’s health beliefs. To “read the whole health story” and make sense of it appears to be a good vantage point for preserving the salutogenic effects of feeling secure and connected to oneself and the world attributed to r/s. It is an endeavour that is ultimately directed at understanding, not at judging the correctness of someone’s health narratives. This may pose a challenge for health promotion, yet it also offers the chance to mitigate healthism and health disparities.
